# Supplementary material for: Comparative chemical analysis of army ant mandibular gland volatiles (Formicidae: Dorylinae)
Source: PeerJ. 2018 Jul 16;6:e5319. doi: 10.7717/peerj.5319 (PMC6052855; doi:10.7717/peerj.5319)

Supplement:

Comparative chemical analysis of army ant mandibular gland volatiles (Formicidae: Dorylinae)

Adrian Brückner*, Philipp O. Hoenle and Christoph von Beeren

* adrian.brueckner@gmail.com

Figure S1 Individual based cluster analysis (UPGMA on merged $d_{A,B}$). Abbreviations: *Eciton burchellii foreli* Mayr 1886 (= Eb), *E. dulcium crassinode* Borgmeier 1955 (= Ed), *E. hamatum* Fabricius 1781 (= Eh), *E. lucanoides conquistador* Weber 1949 (= El), *E. mexicanum* s. str. Roger 1863 (= Em), *E. vagans angustatum* Roger 1863 (= Ev) and *Nomamyrmex esenbeckii wilsoni* Santschi 1920 (= No).

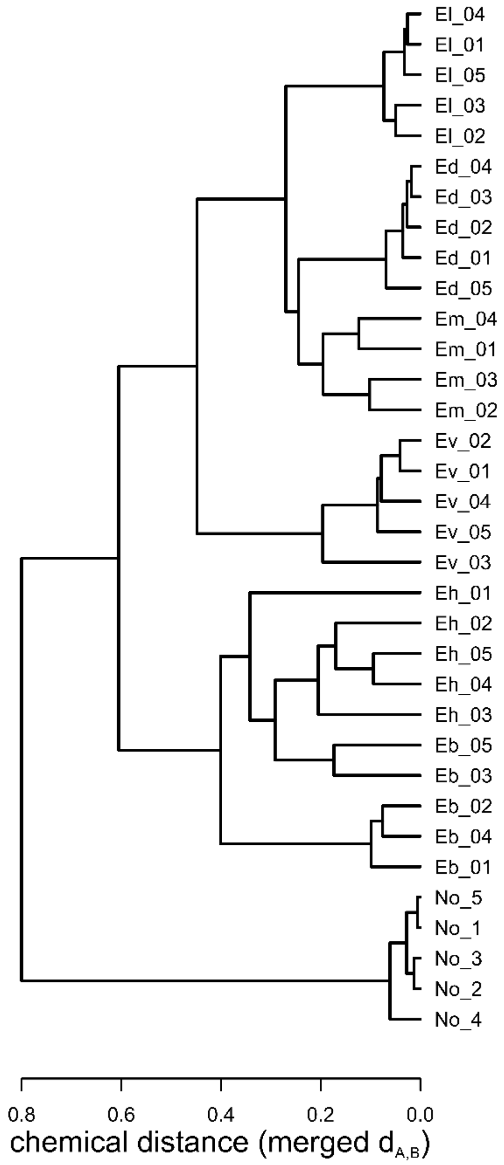

Figure S2. Collection sites and collection dates of *Eciton* major workers and *Nomamyrmex* workers used for chemical analyses. Workers were chosen hapharzdly from the collection by taking specimens from as many colonies as possible for a given species. For instance, in *E. dulcium* (2 collection events) we took 3 majors from the collection event on the 11th of April and 2 majors from the collection event on the 29th of March. The collection map was created by importing GPS data into Google MyMaps. © Google MyMaps

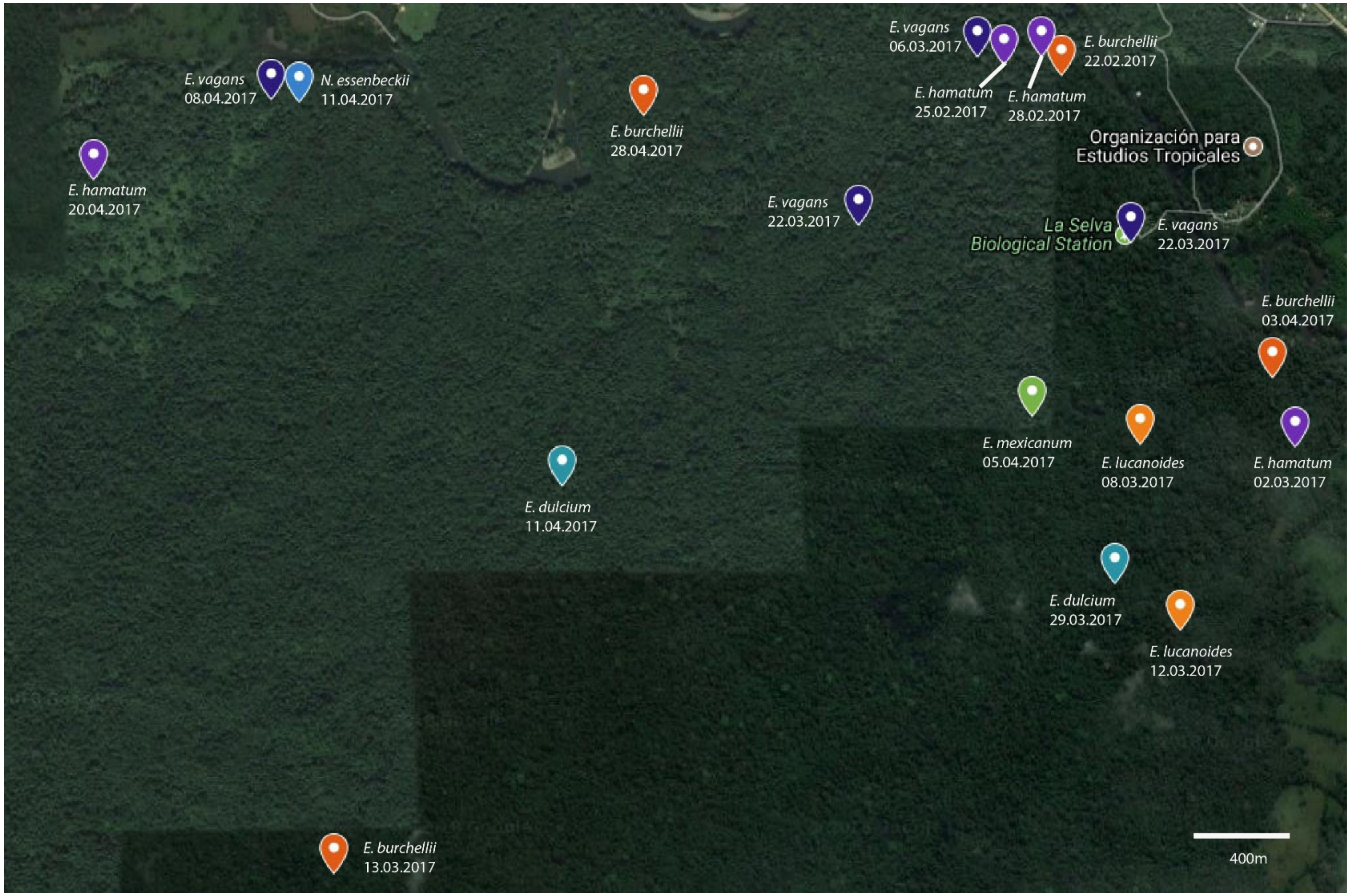

Supplement: Supplemental Information 1 — Individual based cluster analysis (UPGMA on merged dA,B). Abbreviations: Eciton burchellii foreli Mayr 1886 (= Eb), E. dulcium crassinode Borgmeier 1955 (= Ed), E. hamatum Fabricius 1781 (= Eh), E. lucanoides conquistador Weber 1949 (= El), E. mexicanum s. str. Roger 1863 (= Em), E. vagans angustatum Roger 1863 (= Ev) and Nomamyrmex esenbeckii wilsoni Santschi 1920 (= No). [file peerj-06-5319-s001.pdf]
